# A multicenter study to assess efficacy, safety, and tolerability of ropeginterferon alfa-2b-njft in patients with essential thrombocythemia in the US and Canada: EXCEED-ET trial

**DOI:** 10.3389/fmed.2025.1548590

**Published:** 2025-04-17

**Authors:** Lucia Masarova, Brandi N. Reeves, Firas El Chaer, Lynda Foltz, Tsewang Tashi, Ghaith Abu-Zeinah, Jennifer Lucas, Anna B. Halpern, Dawn Maze, Albert Qin, Hana Safah, Fengshuo Lan, Casey L. O'Connell, Swati Goel, Lindsay Rein, Bruno Fang, Joan How, Sunil Babu, Zhuoyan Li, Sonia Cerquozzi, Stephen T. Oh, Anthony M. Hunter, Nikolai Podoltsev, Pankit Vachhani, Abdulraheem Yacoub, Julia M. Cunningham, Christopher Hillis, Salman Otoukesh, Oleh Zagrijtschuk, Henry Castro, Prithviraj Bose

**Affiliations:** ^1^Department of Leukemia, Division of Cancer Medicine, The University of Texas MD Anderson Cancer Center, Houston, TX, United States; ^2^Division of Hematology, Department of Medicine, Blood Research Center, Lineberger Comprehensive Cancer Center, The University of North Carolina at Chapel Hill, Chapel Hill, NC, United States; ^3^Division of Hematology and Oncology, Department of Medicine, The University of Virginia, Charlottesville, VA, United States; ^4^Division of Hematology, St. Paul’s Hospital, University of British Columbia, Vancouver, BC, Canada; ^5^Huntsman Cancer Institute, University of Utah, Salt Lake City, UT, United States; ^6^Division of Hematology and Medical Oncology, Weill Cornell Medicine, New York, NY, United States; ^7^Medical Oncology and Hematology, Marin Cancer Care, Greenbrae, CA, United States; ^8^University of Washington Medical Center, Seattle, WA, United States; ^9^Princess Margaret Cancer Centre, Temerty Faculty of Medicine, University of Toronto, Toronto, ON, Canada; ^10^PharmaEssentia Corporation, Taipei, Taiwan; ^11^Our Lady of the Lake Medical Center, Baton Rouge, LA, United States; ^12^Stony Brook University Medical Center, Stony Brook, NY, United States; ^13^Keck School of Medicine, University of Southern California, Los Angeles, CA, United States; ^14^Department of Hematology and Oncology, Montefiore Einstein Comprehensive Cancer Center, Albert Einstein College of Medicine, Bronx, NY, United States; ^15^Department of Medicine, Duke University School of Medicine, Durham, NC, United States; ^16^Astera Cancer Care, East Brunswick, NJ, United States; ^17^Dana Farber Cancer Institute/Massachusetts General Hospital, Boston, MA, United States; ^18^Fort Wayne Medical Oncology & Hematology, Fort Wayne, IN, United States; ^19^Greater Baltimore Medical Center, Baltimore, MD, United States; ^20^Division of Hematology and Hematologic Malignancies, University of Calgary, Calgary, AB, Canada; ^21^Division of Hematology, Department of Medicine, Washington University School of Medicine, St. Louis, MO, United States; ^22^Department of Hematology and Medical Oncology, Winship Cancer Institute of Emory University, Atlanta, GA, United States; ^23^Hematology Section, Department of Internal Medicine, Yale School of Medicine, New Haven, CT, United States; ^24^O’Neal Comprehensive Cancer Center at the University of Alabama at Birmingham, Birmingham, AL, United States; ^25^Hematologic Malignancies and Cellular Therapeutics, University of Kansas Medical Center, Kansas, MO, United States; ^26^Division of Hematology-Oncology, Lombardi Comprehensive Cancer Center, Medstar Georgetown University Hospital, Washington, DC, United States; ^27^Department of Oncology, McMaster University, Hamilton, ON, Canada; ^28^Department of Hematology and Hematopoietic Cell Transplantation, City of Hope National Medical Center, Duarte, CA, United States; ^29^PharmaEssentia USA, Burlington, MA, United States; ^30^Everest Clinical Research, Markham, ON, Canada

**Keywords:** ropeginterferon alfa-2b-njft (ropeg), essential thrombocythemia (ET), higher initial dose and accelerated titration (HDAC) regimen, complete hematologic response, molecular response, clinical trial

## Abstract

No new drugs have been approved for essential thrombocythemia (ET) treatment since the anagrelide approval in 1997. Ropeginterferon alfa-2b-njft (ropeg) is approved for polycythemia vera, providing a rationale for its use in ET. Its current dosing schema requires dose up-titrations with 50 mcg every 2 weeks and takes approximately 20 weeks to reach a plateau. The goal of this study is to assess the efficacy and safety of ropeg in ET using a higher initial dose and accelerated titration (HDAC) regimen. This is a single-arm, multicenter study in the US and Canada. Patients with ET receive ropeg at 250 mcg on Day 0, 350 mcg at Week 2, and 500 mcg from Week 4 onward with flexibility of dose adjustment. The primary endpoint is: platelets ≤400×10^9^/L, white blood cells <10×10^9^/L, improvement or non-progression of spleen size or major symptoms, and absence of hemorrhagic or thrombotic events, at months 10 and 13. Secondary endpoints include molecular response, safety and tolerability. A total of 91 patients were enrolled with 77 (84.6%) patients in the US and 14 (15.4%) in Canada. The last patient was enrolled on March 28, 2024. *JAK2*V617F was found in 52 (57.1%) patients while *CALR* and *MPL* mutations in 34 (37.4%) and 5 (5.5%), respectively. As of November 12, 2024, the discontinuation rate was 8.8%. The study results will be available in mid-2025. This study will provide efficacy, tolerability and safety, molecular response and quality of life data that will be critical in assessing ropeg for ET treatment.

**Clinical trial registration:**

ClinicalTrials.gov, identifier NCT05482971.

## Introduction

Essential thrombocythemia (ET) is a Philadelphia chromosome-negative myeloproliferative neoplasm (MPN) associated with thrombocytosis, symptoms, and increased risk of developing thrombo-hemorrhagic events and transformation to myelofibrosis or acute myeloid leukemia (1, 2). Most cases of ET (~55%) carry the constitutively active point mutation in *Janus kinase 2*, *JAK2*V617F. Calreticulin (*CALR*) and myeloproliferative leukemia virus oncogene (*MPL*) mutations occur in approximately 15–24 and 4% of ET patients, respectively (1). Treatment decisions in ET are based upon thrombosis risk and symptoms. Patients at lower thrombosis risk are usually managed with aspirin alone while hydroxyurea (HU) is used as the first-line therapy in high-risk or symptomatic patients (3). HU treatment is associated with adverse events (AEs) including fever, rash, stomatitis, gastrointestinal upset, oral and leg ulcers, and increased risk of non-melanoma skin cancer. Patients receiving HU treatment can become intolerant or resistant to therapy, and HU resistance is associated with an increased risk of disease progression and reduced overall survival (4, 5). Anagrelide, an oral imidazoquinazoline derivative, was approved by the US Food and Drug Administration (FDA) to treat ET in 1997 (3). No new drugs have been approved for ET treatment since. Available data suggests that anagrelide therapy does not increase patient overall survival and is associated with toxicity and possibly increases risk of fibrosis progression on long-term follow up (3). Therefore, there is a strong rationale for developing disease-modifying therapies that can not only control hematologic parameters but also reduce mutational allelic burden and potentially provide a survival advantage in the treatment of ET.

Clinical studies suggest that interferon alfa (IFN-*α*)-based therapies are effective in treating thrombocytosis and leukocytosis in patients with ET or polycythemia vera (PV) (6–8). Recombinant IFN-α has also been shown to reduce *JAK2*V617F allele burden, inhibit disease progression, and prolong event-free survival and overall survival of patients with PV (9–11). It is recommended by the National Comprehensive Cancer Network (NCCN) for the management of ET, but does not have regulatory approval for this indication (12). The AEs and cumbersome dosing schedules of conventional IFNs have been significantly reduced with polyethylene glycol (PEG)-conjugation technologies (8, 13). Ropeginterferon alfa-2b-njft (ropeg) is a novel PEGylated IFN-based, anti-neoplastic agent which was approved in the US in November 2021 for the treatment of PV regardless of prior treatment, becoming the first approved IFN-based therapy for the treatment of a Philadelphia chromosome-negative MPN (14). Ropeg has a favorable *in vivo* pharmacokinetic (PK) profile that renders its dosing once biweekly or monthly, improves drug tolerability, and induces durable complete hematologic response (CHR) in the treatment of PV (15–17). In contrast to a high discontinuation rate of 34% due to AEs at 2 years with prior PEGylated IFN-based therapies (18), only 10% of the patients during the ropeg treatment discontinued due to drug-related events over 5 years (19, 20). The standard ropeg dosing schema favors a low starting dose and slow titration in an effort to maximize tolerability and with this strategy, approximately 20–28 weeks of treatment pass before the maximal dose plateau is reached. The dose-exposure-response profile of ropeg is consistent among different ethnic groups and higher PK exposure increases probability of achieving a CHR and reducing the *JAK2*V617F allele burden in patients with PV (21, 22). The reduction of *JAK2*V617F allele burden or variant allele frequency (VAF) is being recognized as an indicator of treatment effect in MPNs as it is associated with a lower risk of thrombotic events and prolongation of event-free survival or even overall survival (23–25). Hematologic control of thrombocytosis and leukocytosis is also relevant as their elevations are associated with thrombosis and disease progression (1, 26, 27). As compared to traditional low dose and slow titration regimens, a higher initial dose and accelerated dose titration (HIDAT) regimen of ropeg induces a more rapid and greater rate of CHR and molecular remission of *JAK2*V617F in patients with PV (28–30). Given its mechanism of action, ropeg could also potentially provide anti-clonal benefits in ET cases including the ~30% of them driven by mutations of *CALR* or *MPL* (31). Ropeg is currently being evaluated in a global randomized Phase 3 study, SURPASS-ET (NCT04285086), in patients with ET who are resistant or intolerant to HU under the HIDAT regimen (32).

There is a high unmet clinical need for effective, safe and tolerable treatment options for patients with ET, both HU treatment-naïve and pre-exposed. Ropeg may be a suitable therapy with the ability to provide clinical benefits as measured by the durable clinical/hematologic response with the potential to not only reduce thrombotic complications, but also to prevent progression to post-ET myelofibrosis and/or secondary acute myeloid leukemia.

## Methods and analysis

### Study design

**Figure 1:**
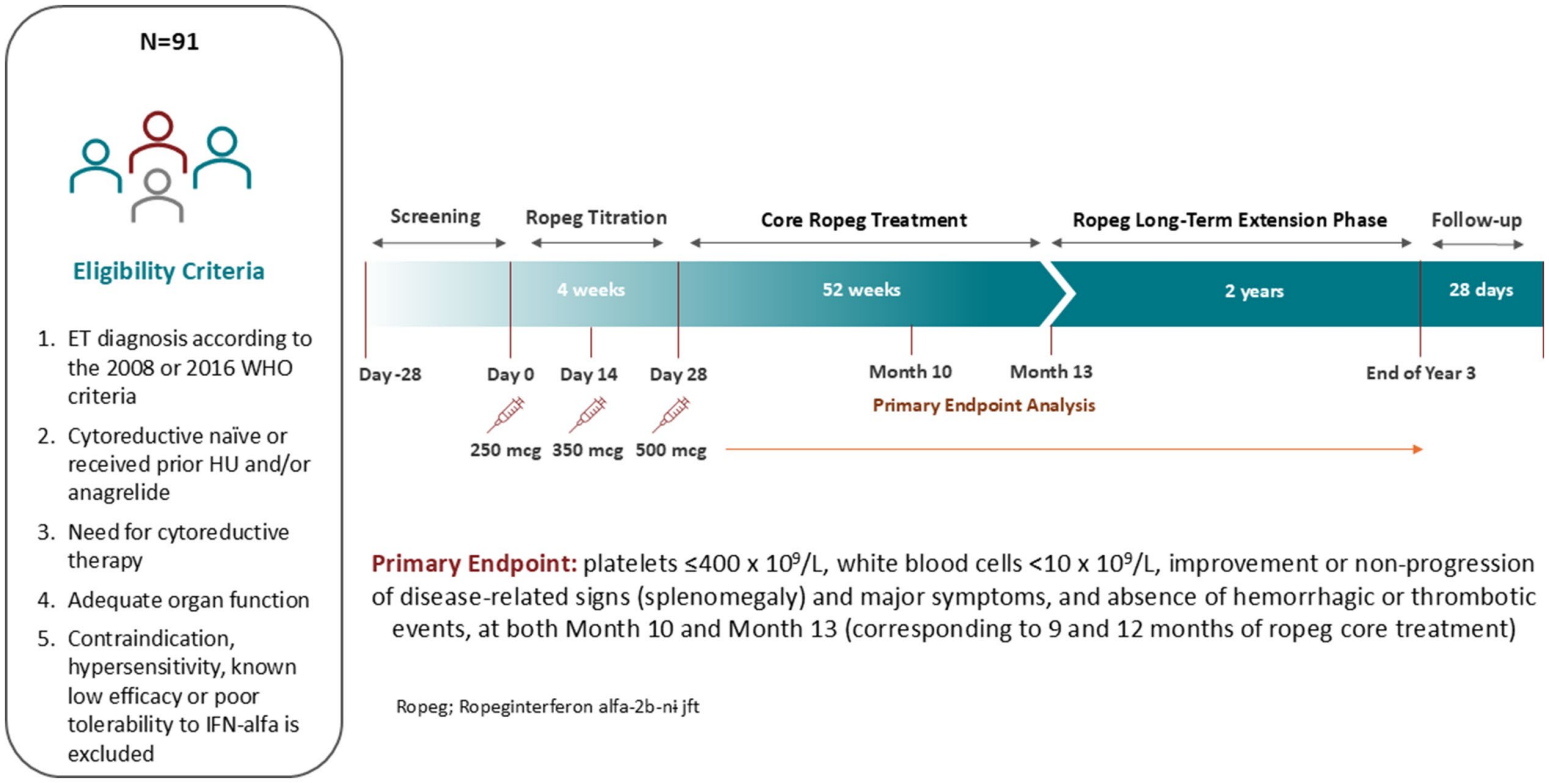
Schematic study design.

This is a single-arm, multicenter study to evaluate the efficacy, safety, and tolerability of ropeg for ET patients in the US and Canada in need of cytoreductive therapy. The indication for cytoreductive treatment for treatment-naïve patients is defined as progressive leukocytosis and/or thrombocytosis, disease-related symptoms (i.e., pruritus, night sweats, fatigue), vasomotor/microvascular disturbances including headache, chest pain or erythromelalgia that are not responsive to aspirin, history of thrombosis at any age, or age > 60 years with *JAK2* mutation. Both treatment-naïve and HU- or anagrelide-pretreated patients are enrolled. The schematic study design is shown in Figure 1.

### Selection of patients

Eligibility criteria include ET diagnosis according to the 2008 or 2016 WHO criteria; adequate organ function defined as bilirubin ≤1.5 × upper limit normal (ULN), prothrombin time or international normalized ratio ≤1.5 x ULN, albumin >3.0 g/dL, alanine aminotransferase ≤2.0 x ULN, aspartate aminotransferase ≤2.0 x ULN, creatinine clearance ≥40 mL/min. Males and females of childbearing potential must agree to use an acceptable form of birth control. Exclusion criteria include any contraindication to IFN-*α* or prior hypersensitivity, known low efficacy or poor tolerability to IFN-α treatment; severe or serious conditions that may affect the participation, compliance and result assessment; history of major organ transplantation; pregnancy or lactation; history of any malignancy within 5 years not curatively treated; and use of any investigational drug <4 weeks prior to the first dose of study drug or on-going effects of prior administration of any investigational drug. The key inclusion and exclusion criteria are listed in Table 1.

**Table 1:** Eligibility criteria of the study.

Inclusion criteria	Exclusion criteria
Age ≥ 18 years	Patients who stopped prior interferon alfa (IFN-*α*) therapy due to low efficacy or poor tolerability.
Diagnosis with ET according to the World Health Organization (WHO) 2016 Criteria	Any contraindications or hypersensitivity to IFN-α and/or its excipients
Cytoreductive treatment-naïve, or pre-exposed to hydroxyurea (HU) and/or anagrelide: Cytoreductive-naïve patients must be in need of cytoreductive treatment, defined as having at least one of the following: i. progressive leukocytosis and/or thrombocytosis ii. disease-related symptoms (i.e., pruritus, night sweats, fatigue) iii. Vasomotor/microvascular disturbances not responsive to aspirin (including headache, chest pain or erythromelalgia, etc.) iv. high risk (history of thrombosis at any age; or age > 60 years with JAK2 mutation) Patients with previous exposure to HU have: i. documented HU resistance or intolerance, or ii. stopped without documented resistance or intolerance due to insufficient blood count control or toxicity	Co-morbidity with severe or serious condition that, in the Investigator’s opinion, would jeopardize the safety of the patient or their compliance with the protocol, including significant cardiac disease such as New York Heart Association Class III-IV congestive heart failure or clinically significant arrhythmias, and pulmonary hypertension
Bilirubin ≤1.5 × upper limit normal (ULN); prothrombin time (international normalized ratio) ≤1.5 x ULN; albumin >3.0 g/dL; alanine aminotransferase (ALT) ≤2.0 x ULN; aspartate aminotransferase ≤2.0 x ULN at screening; except rare hereditary conditions, e.g., Gilbert syndrome, if liver damage and other severe underlying disorders can be ruled out	History of major organ transplantation
Creatinine clearance ≥40 mL/min (by Cockcroft-Gault equation)	Pregnant or lactating females
Males or females of childbearing potential, as well as all women <2 years after the onset of menopause, must agree to use an acceptable form of birth control until 60 days following the last dose of the study drug	Documented autoimmune disease (e.g., poorly controlled thyroid dysfunction, idiopathic thrombocytopenic purpura, scleroderma, psoriasis, or any arthritis of autoimmune origin)
Platelet count >450 × 10^9^/L at screening	Pulmonary infiltrates, pneumonia, and pneumonitis at screening that, in the Investigator’s opinion, would jeopardize the patient safety or compliance with the protocol
Both anagrelide-naïve and -pretreated patients are eligible for the study Written informed consent obtained from the patient, and ability of patient to comply with the study requirements	Infections with systemic manifestations, e.g., bacterial, fungal, or viral infections, except well controlled human immunodeficiency virus, hepatitis B and/or hepatitis C viruses at screening.
	Evidence of severe retinopathy (e.g., cytomegalovirus retinitis, macular degeneration) or clinically relevant ophthalmological disorder (due to diabetes mellitus or hypertension)
	History or presence of clinically relevant depression
	Previous suicide attempts or at any risk of suicide at screening, in the judgment of the investigator
	History or presence of clinically significant neurologic diseases
	History of any malignancy within 5 years (except adequately treated non- melanoma skin cancer, prostate cancer having received surgical resection with an undetectable prostate-specific antigen, curative treated in-situ cancer of the cervix, ductal carcinoma in-situ of the breast, or other cancer curatively treated with no evidence of disease for ≥2 years prior to study)
	History of alcohol or drug abuse within the last year
	History or evidence of any other MPN
	Any investigational drug <4 weeks prior to the first dose of ropeg or not recovered from the effect of prior administration of any investigational agent 17.Prior use of Janus kinase inhibitors

### Study treatment

Eligible patients receive ropeg subcutaneously every 2 weeks with the starting dose of 250 mcg on Day 0, 350 mcg at Week 2, and 500 mcg from Week 4 if tolerable. The dose can be adjusted according to safety or tolerability. If 250 mcg leads to toxicities, the following dose reductions are allowed upon discussion and approval by the Sponsor: dose levels −1 (200 mcg), −2 (150 mcg), and − 3 (100 mcg).

Patients entering the study receiving HU or anagrelide must discontinue their treatment prior to the initiation of ropeg therapy on Day 0. After stopping HU or anagrelide, neither drug is allowed to be concomitantly administered with ropeg.

#### Dose reduction and dose interruption

Dose reduction or interruption of ropeg is recommended in patients experiencing AEs. If major intolerance persists after dose reduction or interruption, therapy should be discontinued. These dose changes, date and time must be recorded on the Dosage Administration Record and AE electronic case report form (eCRF) page.

Dose reduction of ropeg is driven exclusively by safety and tolerability. If a certain dose is poorly tolerated and drug-related toxicities arise, the dose must be reduced to the prior dose, or interrupted as follows:

- If a patient has a severe (Grade 3 or 4) toxicity, or a drop in absolute neutrophil count (ANC) to below 0.5 × 10^9^/L, temporary interruption must be implemented until recovery of the condition (i.e., Grade 0 or 1). Treatment re-initiation occurs from the prior lower dose. For example, if a Grade 3 AE occurs at 500 mcg, treatment re-initiation will take place with 350 mcg. If there is an absence of response (platelet count >400 × 10^9^/L) at this decreased dose after 3 months, the dose can be increased to the prior level.
- If a patient has a Grade 2 toxicity, or a drop in ANC to below 0.75 × 10^9^/L but higher than or equal to 0.5 × 10^9^/L, dose reduction without treatment interruption should be considered.
- Grade 1 toxicity does not lead to dose reduction or interruption.

### Assessments and data analysis

Patient visits are scheduled every 14 days (±3 days) during the titration period and the 12-month core study. In the core study, quarterly major assessment visits for the measurement of hematologic parameters and safety are held as in-office visits. Minor assessment visits can be substituted with phone visits under conditions of stable ropeg dose, ability to self-administer, and no need for intensive safety follow-up for AEs. Patients who are benefiting from treatment after the core study can receive ropeg up to a total of 3 years. A safety follow-up, or end of study (EoS) visit takes place 28 days after the end of treatment (EoT) visit.

The primary study endpoint is defined as: platelets ≤400 × 10^9^/L, white blood cells (WBC) <10 × 10^9^/L, improvement or non-progression in disease-related signs (splenomegaly) based on palpation and ultrasound, major symptom improvement or non-progression based on the Myeloproliferative Neoplasm Symptom Assessment Form Total Symptom Score (MPN-SAF TSS), and absence of hemorrhagic or thrombotic events.

Secondary endpoints include hematologic response, durability of response, change of *JAK2*, *CALR* and *MPL* mutation allelic burden, symptomatic improvement, and occurrence of thromboembolic events and progressive disease.

Evaluation of efficacy includes clinical laboratory assessments, allelic burden measurements of *JAK2*, *CALR,* and *MPL* mutations; spleen size measurements, MPN-SAF TSS completion, and optional bone marrow sampling. Hematologic parameters are assessed by local labs and quantitative measurements of *JAK2*, *CALR*, and *MPL* VAF are performed by a central laboratory. They are assessed quarterly during the core study.

Safety endpoints included incidence, causality and intensity of AEs, serious AEs, and discontinuation of study treatment due to an AE, incidence of AEs of special interest (e.g., thrombotic and bleeding events); incidence of abnormalities of vital signs, clinical laboratory tests, results of physical examinations, electrocardiograms, Eastern Cooperative Oncology Group (ECOG) performance status and Hospital Anxiety and Depression Scale (HADS) score. PK parameters including, but not limited to, minimum concentration (Cmin), time to reach the maximum concentration (Tmax), maximum concentration (Cmax), and area under the drug concentration time curve (AUC) will be derived using population PK analysis and the relationship between exposure and efficacy and safety endpoints will be examined using efficacy and response analysis.

Based on the literature review and analysis of available data from a subset of the ropeg-treated patients with PV whose baseline platelets were ≥ 450 × 10^9^/L, we expect that no treatment has minimal effect in inducing durable response and conservatively estimate a 30% durable response rate by Month 13 as measured at Month 10 and 13 according to the modified ELN response criteria with the ropeg treatment. Assuming a distance from this rate to the lower limit of the 95% one-side confidence interval is ≤10%, the study needs 56 evaluable patients. Further assuming a 12% dropout rate, a total of 64 patients are needed for the study. Final analysis of the primary endpoint will be based on the 13-month dataset. No formal hypothesis is planned to be tested for the extension of the study. Only patients who benefit from 13 months of active treatment and are willing to continue the ropeg treatment are enrolled into the extension. Secondary efficacy analyses will be performed for exploratory purposes using descriptive analysis and standard statistical tests. The secondary analyses are intended to be conducted for all patient subgroups.

## Discussion

Recruitment was completed with 91 patients enrolled. 77 (84.6%) patients were enrolled in the US and 14 (15.4%) in Canada with the last patient enrolled on March 28, 2024. The study was initially planned to enroll 64 patients, but 91 eligible patients were enrolled due to rapid enrollment and a high interest level among the investigators and patient community. *JAK2*V617F was found in 52 (57.1%) patients while *CALR* and *MPL* mutations in 34 (37.4%) and 5 (5.5%), respectively. As of November 12, 2024, the discontinuation rate was 8.8%. Therefore, the study will be completed, and results are scheduled to be available in the middle of 2025.

There is a current need for new effective and tolerable therapeutics for ET treatment in the US and Canada, and in particular for therapies that can target neoplastic clones and induce durable hematologic response with the potential to prevent disease progression. HU is commonly used as a first line option, but it has limitations due to known toxicities and lack of disease-modifying effect. There is also a lack of viable second line option since anagrelide is not considered to be a suitable option by many physicians and patients because of the risk for AEs such as cardiovascular toxicity.^3^ IFN-based treatment has a great potential and has shown clinical efficacy in the treatment of ET.^6, 7^ Despite not being formally approved, it is a recommended treatment option for ET by the NCCN, European Leukemia Net [ELN], and other treatment guidelines. In almost all ET trials utilizing IFN therapy, patients experienced the normalization of platelet count and correction of leukocytosis as part of the hematologic responses.^6^ The toxicity associated with initial recombinant IFN preparations was significant, however, leading to a high treatment discontinuation rate. The issue of poor tolerability of IFN-based therapies is addressed by the improved PEGylation technology, as in case of ropeg. The EXCEED ET study will generate valuable data on the efficacy and safety of ropeg for the treatment of patients with ET.

Therefore, the development of a new ET treatment that can target neoplastic clones and induce durable hematologic response with the potential to prevent disease progression is highly needed. This study will contribute to valuable insights into optimizing the dosing strategy that other research efforts are also involved in (33), and together with the ongoing randomized controlled SURPASS-ET study (NCT04285086), help determine the disease-modifying potential of ropeg for the treatment of ET.
